# Evolution of Hemoglobinopathy Prevention in Africa: Results, Problems and Prospect

**DOI:** 10.4084/MJHID.2009.005

**Published:** 2009-11-10

**Authors:** Slaheddine Fattoum

## Abstract

Hemoglobinopathies are a group of inherited hemoglobin disorders. Initially described in the subtropical regions, they are now spread all around the world because of migration. Their high frequency and clinical severity make them a major public health problem mostly in Africa due to the limited resources reserved for the management and prevention of these diseases. Despite considerable advances in the control and management of the hemoglobinopathies, therapeutic approach and follow- up still pose problems because of the major economic and organizational difficulties in the developing countries, particularly in Africa where problems are majored by other factors including social and cultural backgrounds, high consanguinity, as well as the coexistence of infection and malnutrition. Effective prevention programs have been carried out successfully in many European countries concerned by hemoglobinopathies. They should be extended to African regions where hemoglobin disorders account for more than 70% of total hemoglobinopathies in the world. Prevention should remain the major priority of health services to reduce incidence of hemoglobinopathies in Africa. Hereby we present the Tunisian experience that may reflect globally the profile of the prevention evolution of hemoglobinopathies in North Africa.

## Introduction:

Hemoglobinopathies are a group of inherited autosomal recessive hemoglobin disorders, resulting in the homozygous state, in chronic severe anemias. Thalassemias and sickle cell diseases constitute the most monogenic hemoglobin disorders worldwide. Initially described in the tropical and subtropical regions, they are now common all around the world because of migration.

A global epidemiological database for hemoglobin disorders has been established and published recently by the World Health Organization (WHO). Data collected in 229 countries, clearly indicated that hemoglobinopathies constitute a significant health problem in 71% of those countries which include 89% of all births worldwide1.

The prevalence of carriers of abnormal hemoglobin is within the range of 5–7% in the world and the number of new cases of affected infants is estimated at 300 000 per year. Among them 60 000, are born with beta-thalassemia major annually, the remaining (83%) with Sickle Cell Disease (SCD).

## Hemoglobinopathies in Africa:

More than 70% of total hemoglobin disorders are localized in Africa. African population represents less than 10% of the world population but has the highest crude birth rate (39.0‰) as shown in table 1^1^ and also the highest rate of affected conceptions (10.78‰) compared to the one registered in the world (2.73‰).

Among these affected conceptions, sickle cell diseases (SCD) are largely prevalent (10.68‰) by comparison to thalassemia syndromes (0.07‰). In addition, it is also in Africa where we registered the highest percentage of under-5 mortality among affected births. Table 2^1^ shows some practical service indicators highlighting the needs of care and prevention for hemoglobin (Hb) disorders in African sub-regions. Although only 17.8% from the total births are born in Africa, more than 70% of the annual hemoglobin disorders are also born in Africa. Most of them concerned SCD (85%) and beta-thalassemia major (3.5%). In fact the last percentage corresponds to more than 1500 beta-thalassemia patients born annually; this constitutes a heavy burden with regards to the high cost of treatment of thalassemia and the low income in the majority of African countries.

The most concerned regions by beta-thalassemia in Africa are the western Africa and the Northern Africa with respectively 63.88% and 22.17% of the total annual affected conceptions. The major forms of alpha thalassemia are almost absent in Africa.

## In North Africa:

**Figure 1:**
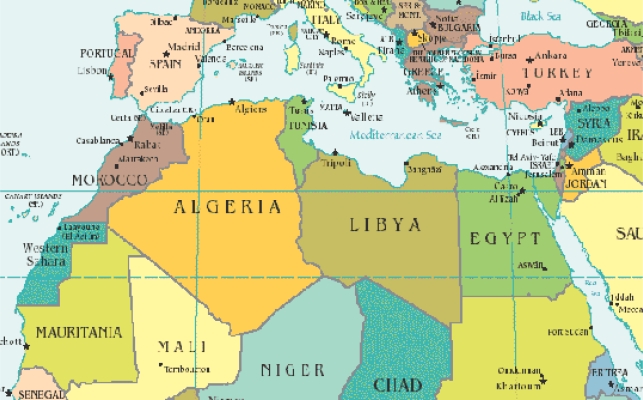
Geographical situation of North African countries

North Africa consists of 5 countries: Mauritania, Morocco, Algeria, Tunisia and Libya (Figure 1) with a total population of 87 millions of inhabitants and a birth rate ranging between 16.8‰ and 40.9‰. The geographical position of this area at the parting of the ways between sub-Saharan African countries and the Mediterranean makes North Africa well concerned by hemoglobinopathies. The inherited hemoglobin disorders are frequently encountered in this population and present a great diversity in the phenotypic expression and genetic aspects.

Many factors may explain, at least partly, this diversity. The most significant factors include the historical context : North Africa witnessed several successive waves of invaders coming from different origins who settled in the region for more or less long periods leading to a large variety of genetic disorders.Ancient social customs, like the high consanguinity rate and endogamy, characterize those populations since centuries, and constitute an additional factor for dissemination of hemoglobinopathies in this area.

### Follow-up conditions

Therapeutic approach in hemoglobin disorders, in particular beta-thalassemia, is based on blood transfusion, iron chelation and specific medical care. Patients present almost the same preoccupations related to growth, schooling, professional integration, social support and financial cares.

Because of the high cost of treatment, difficulties in follow-up affect considerably patients in North Africa due to low-income of concerned population. For these reasons, prevention of hemoglobinopathies becomes a necessity and should be a priority of basic health services to reduce its incidence in each country.

### Requirements for prevention

The prevention program must take into account the epidemiological and molecular data of each concerned country to set up the adequate strategy that includes usually: Sensitization and information about the disease, Population screening and genetic counselling for carriers, and prenatal diagnosis for couples with affected children

## Hemoglobinopathies in Tunisia:

We report here our experience in Tunisia, as an example of a developing country well situated in North Africa. Tunisia occupies the most northerly point of the African continent and covers about 167.000 square km, projecting northward into the Mediterranean Sea, toward Sicily only 86 miles away. It lies approximately midway between the straits of Gibraltar and the Suez Canal. This strategic position in North Africa, makes Tunisia serving a cross road between Europe and Africa and between the Eastern and Western parts of the Arab world.

Berbers were the earliest known inhabitants of the area and racially Tunisia’s population is in the majority, descendants of the union of the Berbers and a large variety of people as Greek, Roman, Vandal, Byzantines, Arab, Spanish, and Turkish who have come to Tunisia since the Phoenicians, first settled in the country 3 000 years ago. Before it got its independence in 1956, Tunisia was a French protectorate throughout 75 years.

As a result of this mixture of races and civilizations, there is no doubt that each of these peoples brought a genetic flow, and had a great influence on the genetic structure of the Tunisians, leading to a high frequency of genetic blood disorders, especially the hemoglobinopathies which represent the most common recessive inherited disorder in Mediterranean population.A recent census gave demographic data of Tunisia as follows^2^: Total population = 9932.4 (x10^3^); Annual natality rate = 16.8 ‰ ; Estimated births per year = 166834.32; Consanguineous marriages = 33%

### Epidemiological data and distribution of main hemoglobinopathies

The presence of the hemoglobinoapathies in Tunisia has been reported as early as 1950^3^. Since then, several surveys were established to determine the frequency of these affections in the country.

**Figure 2:**
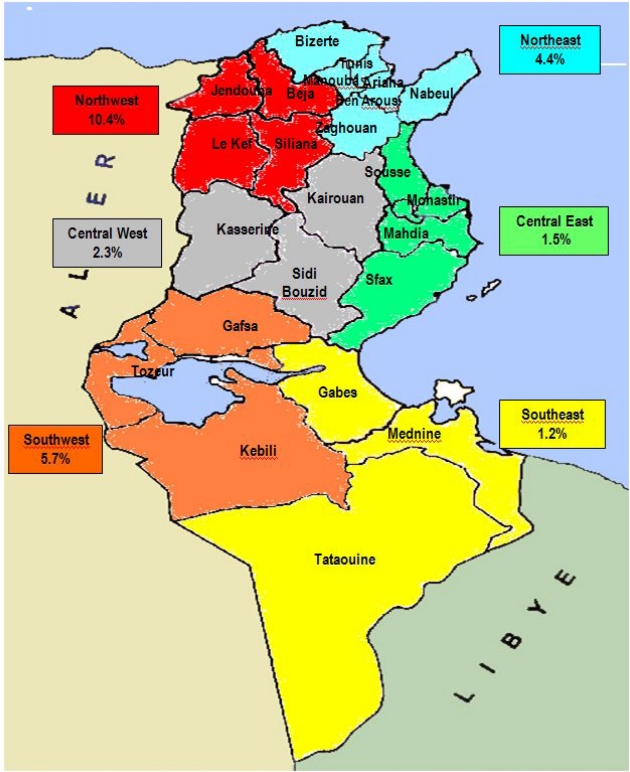
Carrier prevalence of Hb disorders in the different regions of Tunisia

The first results we reported on that field were conducted on Tunisian school children, pregnant women and cord blood samples, noting that beta-thalassemia is the most frequently encountered hemoglobin-pathies among Tunisians^4^. Then through a national screening conducted in the military recruitment centers, findings indicated that the overall frequency of carriers in Tunisia is 4.48% reaching a higher prevalence in the North Western (10.42%) and in the South Western (5.78%) parts of the country. In addition, further investigations allowed to estimate carriers’ prevalence in different regions of the country (Figure 2).

Geographical distribution allowed to identify the most affected areas which are mainly the cities of Tunis, Béja, Jendouba, Le kef, Bizerte, Gafsa, Tozeur, Kasserine, Sousse as well as in the cities of Zaghouan, Nabeul, Sidi Bouzid, Seliana, Kairouan, Sfax and Kebili.

**Figure 3:**
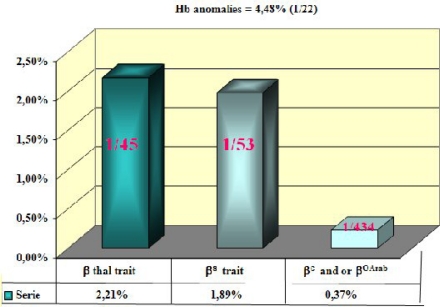
carrier frequency of abnormal haemoglobin

This observation is of a great interest to guide further research and prevention programs. Beta-thalassemia is the predominant hemoglobin abnormality (2.21%) closely followed by the HbS trait (1.89%) (Figure 3).

Finally, the frequency of alpha-thalassemic genes, deduced from the presence of Hb Bart’s in 2628 cord blood samples, has been estimated to 5.48%.^4^

The registered patients are actually over 2400 and the number of at risk couples around 2100 families. The majority of patients (89%) are formed by beta-thalassemic patients, sickle cell patients(SS) and HbS/beta-thalassemia patients (Table 3).

The number of SS patients is unexpectedly much higher than beta-thalassemic patients that would be probably related to the greater tolerance of sickle cell anemia in this country and the natural selection of beta-thalassemia major who, more severely affected, die in early age of life.

Furthermore, according to patient origin, geographical distribution demonstrated once again the higher concentration of beta-thalassemia in the North Western as mentioned above with a newly discovered focus region at Kasserine in the central west of Tunisia where we observed the highest number of beta-thalassemic patients: 27.36% of the total number registered through the country (Table 4).We also note that the most at risk region for sickle cell is the North Western Tunisia (59.70%).

### Molecular basis of beta-thalassemia

Identification of beta-thalassemia mutations is also a pre-requisite for prevention programs before undertaking the prenatal diagnosis offered to at risk couples.The spectrum of beta-thalassemia mutations in the Tunisian population (Table 5) revealed a great molecular heterogeneity with actually 28 different beta thalassemia alleles (6); two common alleles, the codon 39 (C→T) non sens mutation and the beta^+^ IVS1-110 (A→G) mutation accounted for 70% of total beta-thalassemia mutations detected in Tunisia; this undoubtedly facilitates the choice of the adapted strategy in the practice of prenatal diagnosis.

### Prevention program

Epidemiological and molecular data mentioned above are fundamental to approach prevention program. They allow in particular to identify the most at risk regions and the particular spectrum of beta-thalassemia mutations among Tunisians and also the correlations between phenotype and genotype which may have an important influence in the orientation of genetic counselling.

### Prenatal diagnosis

**Figure 4:**
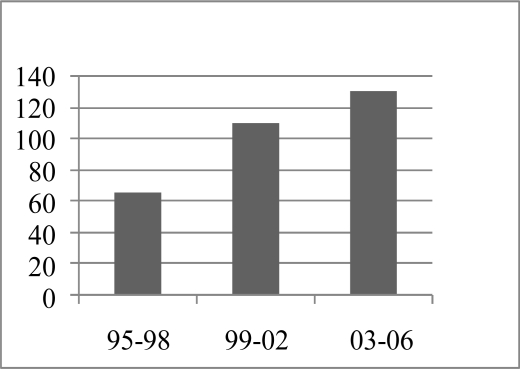
Prenatal diagnosis (PND) among at risk Tunisian couples (1995–2006)

The first setting up of prenatal diagnosis started in 1991 after a transfer of technology based on PCR methods The System was fully functional at the beginning of 1994. Demands of Prenatal diagnosis (PND) followed a relatively good evolution indicating a progressive increase in the number of at risk couples who desire PND. (Figure 4) Between 1997 and 2006 a total of 268 PND were performed revealing 79 affected fetus (29.5%). Termination pregnancy was accepted in 96.5% of cases; refusal was only in 3 cases (3.8%): for religious reason (1 case), delayed diagnosis (2 cases). According to our experience, acceptability of PND depends on genetic information including the pertinence of genetic counselling and the comprehension by families. In addition, usually at risk couples decide more easily for abortion option when fetus is affected by beta-thalassemia major because of the severity of the disease compared to SS fetus or another SCD. This is also true when existence of an affected proband in the family.

Despite a large information and sensitization campaign on hemoglobinopathies, many pregnant women from at risk families do not present or present too late, (after the third trimester of gestation) for prenatal test, thus they lose the prenatal diagnosis benefit.

### Neonatal screening

Neonatal screening started in Tunisia as a pilot study in two maternities in the city of Tunis and aimed at detecting sickle cell disease and beta^0^ thalassemia at early age in order to reduce the under-5 mortality rate in the affected infants. Our experience was successful (Table 6) allowing to take in charge precocely the affected babies and to identify numerous at risk couples to be counseled during their eventual next pregnancies; this pilot program has been extended recently to a high risk region in the North Western Tunisia.

### Premarital screening

Premarital screening was carried out randomly on 53 couples; 18% were detected at risk suggesting the importance of this test to secure at risk couples against affected progenitor. The screening program needs to be supported by public awareness and regular education in concerned populations.

## Discussion:

Hemoglobinopathies constitute a real public Health problem in the large majority of African countries and contribute, at least partly, in the increasing rate of infant mortality and morbidity. In the only North Africa Region, demographic and epidemiologic data allow to estimate the number of new cases of babies who are born with a severe form of hemoglobinoathie at as much as 419 every year in Tunisia, Algeria and Morocco and around 2000/year if we include Mauritania and Libya where carrier rates of abnormal hemoglobin are much higher (Table 7)^5^,^7^,^8^,^9^,^10^,^11^.

We can easily imagine the heavy burden of this large number of patients on health care resources, knowing that the direct cost of the management for one patient is estimated to about 7000DT/year (5000 US $).

In reality the whole problem still lies in the difficulties in the diagnosis of abnormal hemoglobin traits and in the very limited economic resources that do not permit to take in charge correctly the numerous patients already identified. Several affected children die before reaching the 10^th^ year of life. Still this figure is probably understated because of the existence of local concentration of the disease and of the high degree of consanguinity, reaching a rate of 70% in some rural areas. In addition, heterozygote detection should be conducted carefully in these countries where iron deficiency is a major problem for the diagnosis of heterozygous beta-thalassemia since it may mask an elevation of HbA_2_ level. Our experience in Tunisia demonstrates that premarital screening is the best option and should be mandatory in any prevention program for hemoglobinopathies. Thus most at risk couples are identified early for prenatal diagnosis in the first pregnancy and would regularly produce healthy offspring. Unfortunately many at risk couples yet discover their risk only after the birth of their first affected child. In Tunisia, neonatal screening was decided to reach the maximum of concerned families; its efficiency is more evident in population of at risk regions. Success will depend on a good coordination and a good cooperation between parents and medical staff.

Moreover, in the most African countries, data on epidemiology of hemoglobinopathies are scarce, and insufficiently precise to undertake a solid program of control and prevention for these genetic diseases. The few preventive actions conducted in this context remain uncompleted and need to be developed efficiently by opting to the best adapted way, economical and cost-effective.

To improve the situation, public education about thalassemia and sickle cell diseases is of a great importance and should be carried out through periodic meetings addressed to health professionals including doctors and nurses working in the community, and family members. Also, all means of mass media are helpful as well as the sensitization through patient parents’associations that facilitates the contact with families and the diffusion of information through didactic supports (brochures, booklets ect…).

## Conclusions:

Sickle cell diseases, β-thalassemia and other hemoglobinopathies are frequent in Africa. Their distribution varies from one region to another. Western and Northern Africa are the most affected areas. Their clinical severity and the poor conditions of medical care make hemoglobinopathies a real public health problem in each country.

Prevention program at national level, remains the best alternative to control hemoglobinopathies in most concerned countries. It should include:

1. An active sensitization of population, notably among the youth;
2. Integration of hemoglobin study as a mandatory pre-marital test;
3. Providing freely genetic counselling to carriers and at risk couples for hemoglobinopathies;
4. Extend the neonatal screening of hemoglobinopathies to all at risk regions;
5. Maintain updated the national patients register for hemoglobinopathies in the country for prospective prevention actions.

Taking in account that SCD present 83% of total haemoglobin diseases, a recent resolution from UN recognizes SCD as a priority of public health and declares the 19^th^ of June of each year as international day to fight against these affections.

## Figures and Tables

**Table 1:** Demographic data and estimated prevalence of carriers of Hemoglobin disorders and affected conceptions^1^

Population	Demography 2003				% of the population carrying			Affected conceptions (per 1000)			**% of under-5 mortality In Affected births**
	Populatio n (millions)	Crude birth rate (‰)	Annual births (1000s)	Under-5 mortality rate (‰)	Sig-nificant Hb variant	alpha+ thalassaemia	Any variant	Sickle cell disorders	Thalassaemias	Total	
**African population**	586	39.0	22895	168	18.2	41.2	44.4	10.68	0.07	10.74	**6.4**
**World population**	6217	20.7	128814	81	5.2	20.7	24.0	2.28	0.46	2.73	**3.4**

**Table 2:** Indicators of annual service needs for hemoglobin disorders^1^

**REGION**	**Annual affected conceptions**			
**-**	**Sickle cell disorders**	**beta thal**	**alpha thal**	**Total annual disorders**
**African Region**	**233 289**	**1 520**	**11**	**234 819**
Northern Africa	181	337	0	627
Western Africa	167 224	971	0	9 622
Middle Africa	40 688	27	0	4 184
Eastern Africa	25 184	183	11	6 974
Southern Africa	11	2	0	1 487
**World**	**276 168**	**42 409**	**13 466**	**332 043**

**Table 3:** Registered patients (1980–2006)

	**Nb of Patients**	**Nb of CAR**

**b thal maj**	**742**	**637**
**SS patients**	**925**	**830**
**S/b-thal**	**480**	**420**

CC	35	35
OO	5	6
SC	76	68
SO_Arab_	43	37
C/b-thal	34	27
O/b-thal	23	19
HbH	14	11
other (SD, CO,etc….)	17	16

**Total**	**2394**	**2106**

**Table 4:** Distribution of the main major hemoglobinopathies. Figure legend: GT-global territory, NE-north east, NW-north west, CE-central east, CW-central west, SE-south east, SW-south west.

	**β-thalassemia major**		**Sickle cell disease**	

	**Nb**	**%**	**Nb**	**%**
GT	37	4.99	130	8.52
NE	129	17.39	297	19.46
**NW**	268	**36.12**	911	**59.70**
CE	71	9.57	45	2.95
SE	9	1.21	13	0.85
**CW**	203	**27.36**	37	**2.42**
SW	25	3.37	93	6.09
Total	742	100.00	1526	100.00

**Table 5:** Spectrum of β-thal mutations among Tunisians (Total chromosomes studied = 470)

**Mutation**	**Type**	**Frequency**

cd39 (C-->T)	β°	49.0%
IVS1-110 (G-->A)	β+	21.0%
IVS1-1(G-->A)	β°	4.5%
cd44 (−C)	β°	3.8%
IVS 1-2(T-->G)	β°	3.0%
cd30 (G-->C)	β+	3.2%
IVS 2- 745 (C-->G)	β°	2.6%
cd6(−A)	β°	2.6%
IVS1-5 (G-->A)	β+	1.5%
−87 (C-->G)	β+	1.7%
IVS1-5 (G-->C)	β+	1.0%
−30 (T-->A)	β+	0.8%
cd25/26 (+T)	β°	0.6%
IVS 1-6 (T-->C)	β+	0.6%
IVS2-1(G-->A)	β°	0.6%
cd5 (−CT)	β°	0.4%
IVS 2-848 (C-->A)	β+	0.4%
IVS2-849 (A-->C)	β+	0.4%
cd8 (−AA)	β°	0.2%
cd47 (+A)	β°	0.2%
cd106/107 (+G)	β°	0.2%
uniditified		1.9%
Total		100%

**Table 6:** NeoNatal Screening of Hemoglobinopathies

**Pilot study Results**						
	Samples studied (19 months)	Minor Forms		Major Forms		
		AS	other min forms	Thal maj	Sickle cell syndromes	Total maj forms
**Nb**	13000	260	351	6	5	14
Abnormal Hb Ratio From total live births		1/50	1/37	1/2166	1/2600	1/928

**Table 7:** Carrier frequencies of abnormal hemoglobin in North African Countries

**Country**	**Global frequency %**	**% Carrier**				**References**
		β-thal	HbS	HbC	Other (HbO Arab, HbD, ..)	
Algeria	3.50	1-60-2.0	0.70	0.40	-	[7], [8]
Libya	14.13	7.77	4.51	-	1.85	[9]
Mauritania	16.60	2.53	8.71	3.00	2.36	[10]
Marocco	2.61	0.95	0.59	1.07	-	[11]
Tunisia	4.48	2.21	1.89	0.23	0.15	[5]
